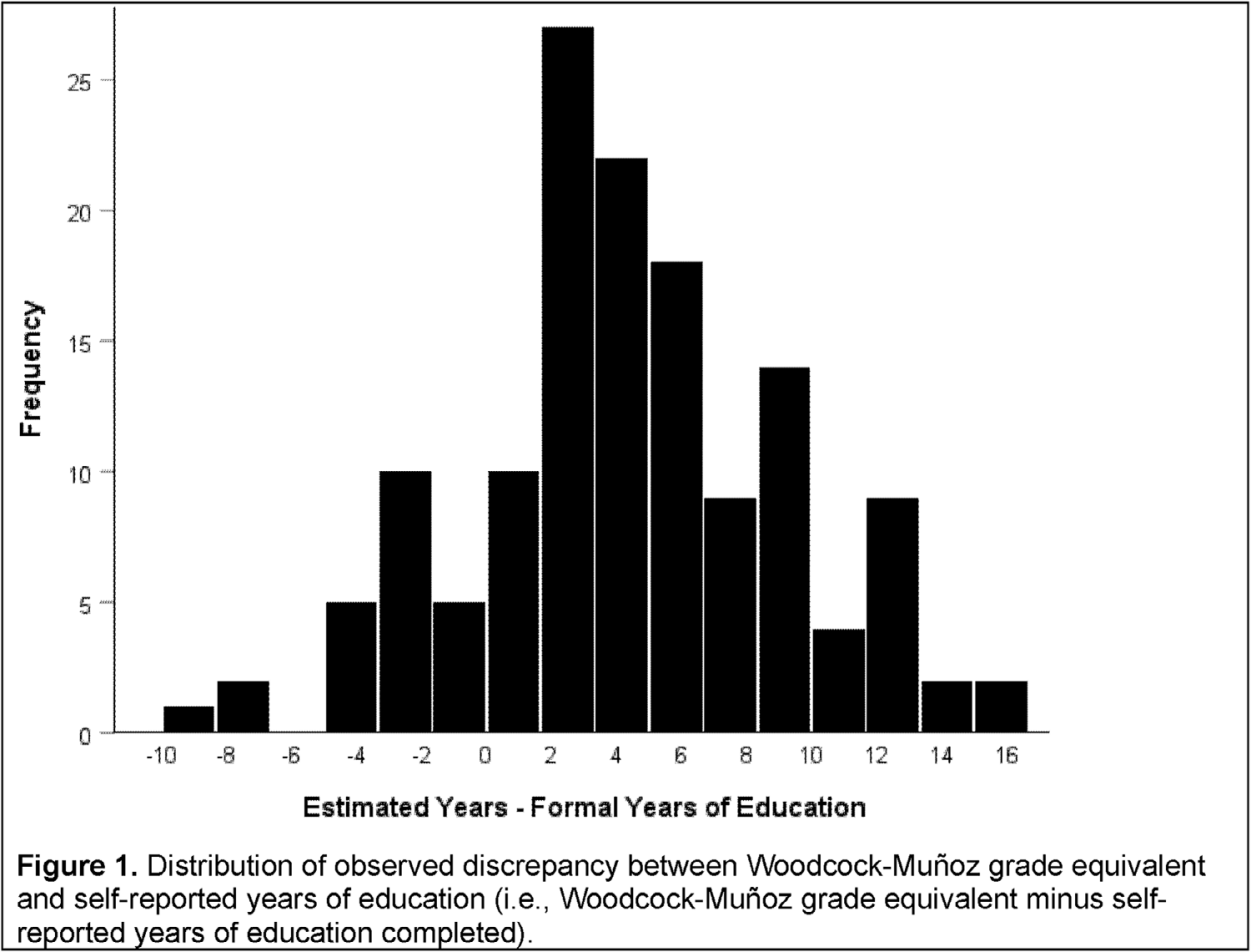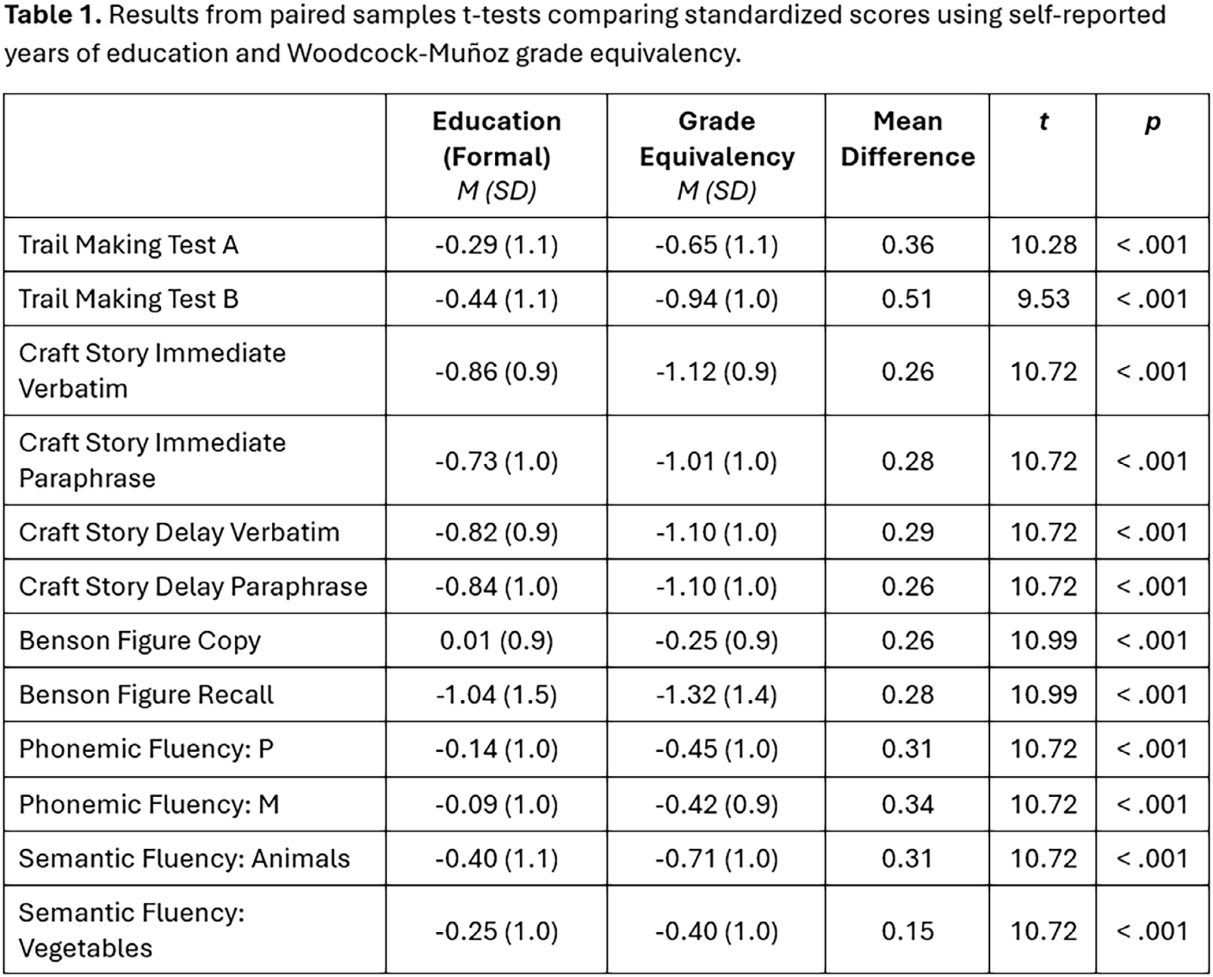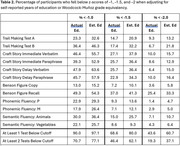# Self‐Reported Education vs. Grade‐Equivalent Reading Level: Potential Implications for Normative Referencing of Cognitive Tests in Spanish‐Speaking U.S. Latinos

**DOI:** 10.1002/alz70857_107149

**Published:** 2025-12-26

**Authors:** Kate Ruiz, Melanie L. Johnson, Diane M. Jacobs, Britney Escobedo, Jocelyn Vargas, Christina Gigliotti, Emily A. Little, Guerry M. Peavy, Zvinka Z. Zlatar, David P. Salmon

**Affiliations:** ^1^ University of California, San Diego, La Jolla, CA, USA; ^2^ Shiley‐Marcos Alzheimer's Disease Research Center, San Diego, CA, USA; ^3^ San Diego State University, San Diego, CA, USA; ^4^ Department of Neurosciences, University of California San Diego, La Jolla, CA, USA; ^5^ Alzheimer's Disease Cooperative Study (ADCS), University of California, San Diego, La Jolla, CA, USA; ^6^ UCSD Shiley‐Marcos Alzheimer's Disease Research Center, La Jolla, CA, USA; ^7^ Alzheimer's Disease Cooperative Study, University of California San Diego, La Jolla, CA, USA; ^8^ Shiley‐Marcos Alzheimer's Disease Research Center, University of California, San Diego, La Jolla, CA, USA; ^9^ Department of Neurosciences, University of California San Diego, San Diego, CA, USA; ^10^ University of California San Diego, Department of Neurosciences, La Jolla, CA, USA; ^11^ Alzheimer's Disease Cooperative Study, La Jolla, CA, USA; ^12^ Shiley‐Marcos Alzheimer's Disease Research Center, University of California, San Diego, CA, USA

## Abstract

**Background:**

Normative references for neuropsychological tests commonly adjust for years of education. However, quality of formal education varies widely, and additional education may be obtained outside of the classroom setting. Therefore, years of education may inadequately reflect full educational achievement. Grade‐equivalency scores from tests of single‐word reading provide additional information about learning experience and may alter interpretation of test results (Manly et al., 2002). Therefore, we explored the impact of adjusting cognitive test results for years of education vs. estimated grade equivalency in Spanish‐speaking U.S. Latinos.

**Method:**

140 Spanish‐speaking Latinos with subjective cognitive decline were referred by a community‐based neurologist for objective cognitive testing. Participants were aged 47‐88 (M=70.6, SD=8.0) with an average of 9.1 self‐reported years of education (SD=4.5, range=0‐20). Participants completed a cognitive screening battery using tests from the National Alzheimer's Coordinating Center Uniform Data Set Neuropsychological Battery (UDS3‐NB). The Woodcock‐Muñoz Letter‐Word Identification Test was used to estimate grade‐equivalency. Standardized scores (Z‐scores) that adjusted for age, sex, language of testing, and either self‐reported years of education or Woodcock‐Muñoz grade‐equivalency were obtained for each cognitive test using the UDS3‐NB Latino Norms Calculator (Marquine et al.,2023). The two sets of Z‐scores were compared using paired samples t‐tests.

**Results:**

Grade equivalency estimated by the Woodcock‐Muñoz (M=13.5, SD=4.6; range=1.7‐18) was significantly higher than self‐reported years of education (M=9.1, SD=4.5; t [139]=10.9, p < .001). Figure 1 shows the distribution of the discrepancy between grade equivalency and self‐reported educational attainment. For all neuropsychological tests, adjusted Z‐scores derived using grade equivalency were significantly lower than those derived using years of education (see Table 1). The percentage of participants who fell below z‐score cutoffs of ‐1, ‐1.5, or ‐2 was greater when adjusting for grade‐equivalency than when adjusting for years of education (Table 2).

**Conclusions:**

Education‐adjusted norms appeared to underestimate level of impairment in Spanish‐speaking U.S. Latinos who presented to a neurologist with cognitive complaints. Self‐reported years of education may not adequately reflect the educational experiences of this diverse group. Although additional validation is needed, these results indicate caution is warranted when interpreting education‐adjusted test scores for Spanish‐speaking U.S. Latinos with lower levels of education.